# Study of the Interactions of Bovine Serum Albumin with the New Anti-Inflammatory Agent 4-(1,3-Dioxo-1,3-dihydro-2*H*-isoindol-2-yl)-*N′*-[(4-ethoxy-phenyl)methylidene]benzohydrazide Using a Multi-Spectroscopic Approach and Molecular Docking

**DOI:** 10.3390/molecules22081258

**Published:** 2017-07-27

**Authors:** Tanveer A. Wani, Ahmed H. Bakheit, Abdul-Rahman A. Al-Majed, Mashooq A. Bhat, Seema Zargar

**Affiliations:** 1 Department of Pharmaceutical Chemistry, College of Pharmacy, King Saud University, P.O. Box 2457, Riyadh 11451, Saudi Arabia; abujazz76@gmail.com (A.H.B.); almajed99@yahoo.com (A.-R.A.A.-M.); mabhat@ksu.edu.sa (M.A.B.); 2 Department of Biochemistry, College of Science, King Saud University, P.O. Box 22452, Riyadh 11451, Saudi Arabia; szargar@ksu.edu.sa

**Keywords:** HSA, BSA, serum albumin, thalidomide, lipophilic thalidomide derivative, fluorescence, quenching, 6P

## Abstract

The lipophilic derivative of thalidomide (4-(1,3-dioxo-1,3-dihydro-2*H*-isoindol-2-yl)-*N′*-[(4-ethoxyphenyl)methylidene]benzohydrazide, **6P**) was synthesized to enhance its characteristics and efficacy. Earlier studies have proved the immunomodulatory and anti-inflammatory effects of **6P**. In this study the interaction between bovine serum albumin (BSA) and **6P** was studied using a multi-spectroscopic approach which included UV spectrophotometry, spectrofluorimetry and three dimensional spectrofluorometric and molecular docking studies. Static quenching was involved in quenching the fluorescence of BSA by **6P**, because a complex formation occurred between the **6P** and BSA. The binding constant decreased with higher temperature and was in the range of 2.5 × 10^5^–4.8 × 10^3^ L mol^−1^ suggesting an unstable complex at higher temperatures. A single binding site was observed and the the site probe experiments showed site II (sub-domain IIIA) of BSA as the binding site for **6P**. The negative values of ∆G^0^, ∆H^0^ and ∆S^0^ at (298/303/308 K) indicated spontaneous binding between **6P** and BSA as well as the interaction was enthalpy driven and van der Waals forces and hydrogen bonding were involved in the interaction. The docking results and the results from the experimental studies are complimentary to each other and confirm that **6P** binds at site II (sub-domain IIIA) of BSA.

## 1. Introduction

Thalidomide and its various derivatives are used as prophylactic drugs in the case of autoimmune diseases due to their immunomodulatory and anti-inflammatory properties [1]. Chemically thalidomide is (α-*N*-phthalimidoglutarimide) and was indicated as a sedative in 1954, however due to its teratogenic effects it was withdrawn and later reintroduced and approved for use in nodosum leprosum and in multiple myeloma [2]. Recent studies have indicated the usefulness of thalidomide in prostate cancer, Crohn’s disease, lupus erythematosus, chronic host-versus-graft disease, HIV-associated oral ulcers, rheumatoid arthritis and Behcet’s disease [3,4,5]. In several animal studies it has been shown to suppress various inflammatory markers such as TNF-α, IL-1β, IL-6, and NO, thus relieving inflammation and pain [6,7,8,9]. To overcome the teratogenic, peripheral neuropathic and other adverse events caused by thalidomide several structural analogues were synthesized and these exhibited less toxicity and were highly potent. Structural modification of thalidomide has produced potent inhibitors of TNF-α [10,11].

**Figure 1:**
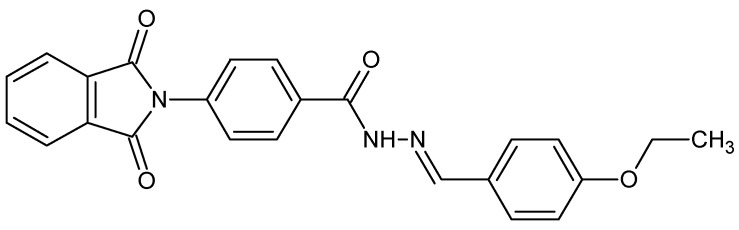
Chemical structure of the lipophilic thalidomide derivative (4-(1,3-dioxo-1,3-dihydro-2*H*-isoindol-2-yl)-*N′*-[(4-ethoxyphenyl)methylidene]benzohydrazide (**6P**).

In a similar fashion the lipophilic thalidomide derivative (4-(1,3-dioxo-1,3-dihydro-2*H*-isoindol-2-yl)-*N′*-[(4-ethoxyphenyl)methylidene]benzohydrazide (**6P**, Figure 1) was synthesized by modifying the thiomorpholine group by introducing hydrophobic moieties to enhance its lipophilic characteristics and anti-inflammatory properties [12]. A decrease in the inflammation markers IL-6, IL-17, TNF-α, NF-κB, GITR, STAT-3, COX-2 and iNOS was observed after treatment with **6P** in a mouse model (lung inflammation) which was further corroborated by the histopathological studies. Anti-inflammatory mediators IL-10 and IL-4 showed an increase post-treatment with **6P** suggesting potent anti-inflammatory agent effect of **6P** in the carrageenan-induced lung inflammation. Further, histopathological examination also confirmed the potent anti-inflammatory effects of the compound **6P**. Compound **6P**, being a small molecule, has the capability to bind to various targets like transcription factor NF-κB p65, 1MY5 PDB-structure, etc., thus increasing its anti-inflammatory potential [12].

Serum albumin (SA) is the major protein involved in the transport of drug ligands, thus affecting the ADME (absorption, distribution, metabolism and excretion) of these drugs [13,14,15]. Thus the first step in evaluation of the pharmacokinetics of these drug ligands is to investigate its affinity towards SA [16]. BSA being the model protein due to its structural resemblance to HSA and its low-cost and obtainability was used to study the interaction between the BSA and **6P**. The biophysical interaction of **6P** with BSA was studied using multi-spectroscopic studies along with molecular docking [15,17,18]. The study will provide a great insight regarding the pharmacokinetic behavior of the **6P** in-vivo.

## 2. Results and Discussion

### 2.1. Study of Conformational Changes in BSA with UV-Visible Spectroscopy

**Figure 2:**
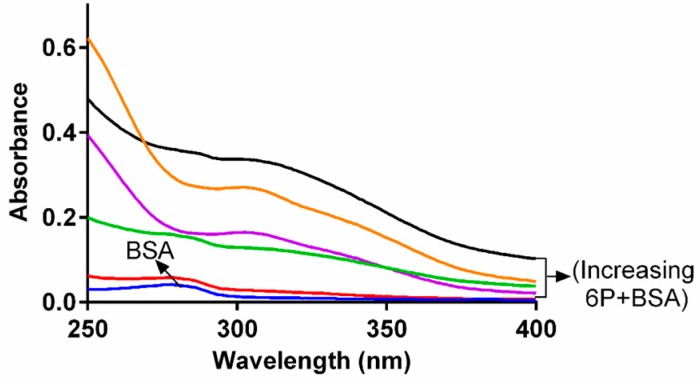
Spectra of BSA in presence and absence of **6P**.

The structural changes and the complex formation within the **6P** and BSA was studied with the help of absorption spectroscopy [19]. The spectra of BSA alone as well as with increasing concentrations of **6P** are presented in the Figure 2. The band at 280 nm produced by the BSA represents the π → π transition because of amino acids present in it. The absorption intensity of BSA increased post **6P** addition, indicating that the BSA molecules were associated with **6P** and formed a **6P**-BSA complex. Further, the maximum absorbance peak shifted slightly towards a lower wavelength. These two results, i.e., shifting of the maximum absorbance and an increase in the absorption intensity together evidently inferred the presence of an interaction between **6P** and BSA and also indicated a change in the conformation of BSA.

### 2.2. Fluorescence Quenching of BSA

**Figure 3:**
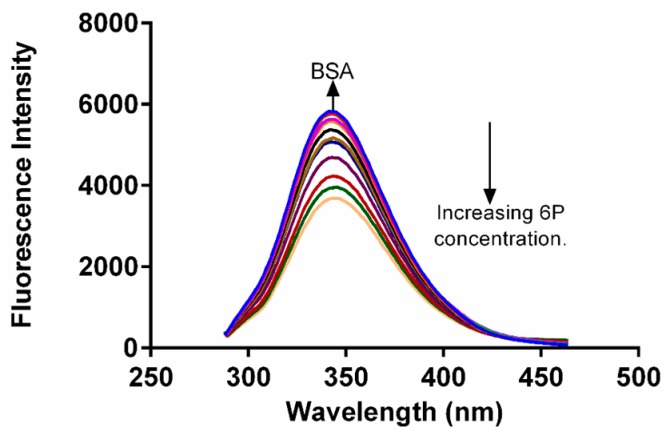
Spectra for fluorescence quenching of BSA in the presence and absence of **6P** at 25 °C.

In drug binding studies, fluorescence quenching methodology plays a very important role in exploration of the interaction between ligand and proteins [20]. The fluorescence spectra of BSA were obtained in absence and in the presence of different concentrations of **6P** (Figure 3). A decrease in the FI of BSA was observed as the concentration of **6P** was increased with a slight change in the λ emission of BSA. This alteration in the λ emission indicated a slight change in the fluorophore microenviroment during the interaction of **6P** and BSA.

### 2.3. Analysis of Fluorescence Quenching and Mechanism

The two processes by which quenching can occur are static quenching and dynamic quenching. In the case of static quenching formation of a complex between the ligand and protein takes place and the complex formed is non-fluorescent in nature. In the case of dynamic quenching the fluorophore and the quencher interact with one another during the course of excitation. The static and dynamic quenching can also be differentiated on the basis of their dependence on temperature and viscosity. In addition to these two parameters they can also be distinguished from each other on the basis of their life time measurements. At higher temperatures in dynamic quenching there is an intensification of diffusion and collisional quenching resulting in a higher quenching constant. A similar increase in temperature in case of static quenching causes the reverse effect on the quenching constant. The mechanism of quenching involved can be determined with help of Stern-Volmer equation:

(1) $$\frac{F}{F_{0}}=1+K_{\mathrm{sv}}\left[Q\right]=1+K_{q}{\;\tau }_{0}\;\left[Q\right]$$

**Figure 4:**
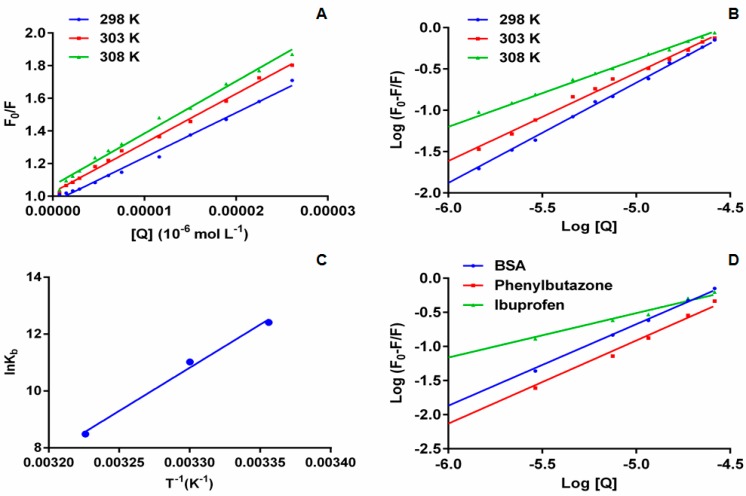
(**A**): The stern–Volmer curves for the quenching of BSA by **6P** at 298/303/308 K; (**B**): The plot of log[(F_0_ − F)/F] versus log[Q] for quenching process of **6P** with BSA at 298/303/3008 K; (**C**): Van’t Hoff plots for the binding interaction of **6P** with BSA; (**D**): The plot of log[(F_0_ − F)/F] versus log[Q] for quenching process of **6P** with BSA in presence of site markers phenylbutazone and ibuprofen at 298 K.

In the above equation F and F_0_ represent the FI of BSA in presence and absence of **6P**; K_sv_, K_q_, [Q] represent the Stern-Volmer constant, bimolecular quenching rate constant and quencher concentration respectively; τ_0_ is the lifetime of fluorophore in absence of quencher and is valued to be 10^–8^ in case of biopolymers. The obtained K_sv_ and K_q_ values are presented in Table 1 (Figure 4A). In addition to K_sv_, bimolecular quenching rate constant also assists to determine the type of quenching involved in the process.

An increase in the K_sv_ and K_q_ values was observed indicating the possibility of involvement of dynamic quenching. The rate constant for scattering collision quenching can attain a maximum value of 2 × 10^10^ L M^−1^ S^−1^. The obtained K_q_ values are much higher than 2 × 10^10^ L M^−1^ S^−1^ indicating the formation of non- fluorescent complex (static quenching) amongst **6P** and BSA.

Although there is a difference between the two quenching mechanisms, the interaction of **6P** with BSA can be further explained from the UV absorption spectra of BSA in presence of **6P**. In the case where dynamic quenching were involved there would had been no change in the absorption spectrum of BSA in the presence of **6P** as only the excitation state of the quencher is affected, with no effect on the UV-absorption spectra of BSA, whereas in the case of static quenching formation of a complex occurs between the ligand and the protein causing changes in the UV-absorption spectra of BSA in the presence of ligands. According to the UV spectra complex formation (i.e., static quenching) occurred between BSA and **6P** which was further corroborated with the obtained high K_q_ values [20,21,22,23].

### 2.4. Binding Constant and Binding Modes

To determine the binding constant (K_b_) and binding site number (n) double log regression curve is used (Figure 4B) [23]. It is assumed that one or more than one binding sites are available on the protein molecule to bind the ligand. The K_b_ and n are calculated from the intercept and slope of the plotted double log regression curve (Table 2):

(2) $$\log\frac{\left(F_{0}-F\right)}{F}={\log\;K}_{b}+n\;\log\left[Q\right]$$

The strength of the binding interaction is inferred from the values of K_b_—the higher the value of K_b_ the stronger the binding between the protein and the ligand. The results show a decrease in the K_b_ values with an increase in temperature, demonstrating the instability of the **6P**-BSA complex at higher temperatures. It also suggests that static quenching is involved in the interaction between **6P** and BSA. The number of binding sites involved in the interaction of **6P** and BSA at all the studied temperatures were approximate equal to 1. A correlation coefficient (*r*^2^) of >0.99 at all the three studied temperatures indicates that the interaction between **6P** and BSA followed a binding model based on the double log regression method.

Further, phenylbutazone and ibuprofen were taken as site-specific probes to locate the binding site I and II of BSA, respectively, and their interaction with **6P**. To obtain this varied concentrations of **6P** were added to fixed concentrations of BSA protein and site probe marker. The fluorescence spectra of the resulting solutions were obtained at room temperature with an excitation wavelength of 280 nm. The deviations in the fluorescence spectra were utilized to obtain the binding constants (Figure 4C). For the **6P**-BSA-phenylbutazone system the binding constant was found out to be 1.26 × 10^5^ L·mol^−1^ and for the **6P**-BSA-ibuprofen system the binding constant was found to be 5.49 × 10^2^ L·mol^−1^. These binding constants when compared to the **6P**-BSA system 2.50× 10^5^ L·mol^−1^ showed a remarkable decrease in the presence of ibuprofen whereas the K_b_ values showed little effect in the presence of phenylbutazone. These results indicate site II (sub-domain IIIA) as the binding site for **6P** [24].

### 2.5. Thermodynamic Parameters and Binding Forces

Drugs bind to protein molecules with the help of different types of binding forces, including van der Waals forces, hydrogen bonding, electrostatic interactions and hydrophobic interactions. Van’t Hoff’s equation is used to obtain the thermodynamic parameters. The signs of these forces (positive or negative) and the amount of these forces helps in determination of types of interaction involved within the ligand and the protein. Van’t Hoff equation is given as follows:

(3) $${\mathrm{lnK}}_{b}=-\frac{\Delta H^{0}}{\mathrm{RT}}+\frac{\Delta S^{0}}{R}$$

(4) $$\Delta G^{0}=\Delta H^{0}-T\Delta S^{0}=-{\mathrm{RTln}\;K}_{b}$$

where ∆G^0^, ∆H^0^ and ∆S^0^ represent the change Gibbs free energy, enthalpy and entropy, respectively, and R and K_b_ represent the universal gas constant and binding constant, respectively. The negative sign of the values for ∆H^0^ and ∆S^0^ represents the contribution of van der Waals forces and/or hydrogen bonding. The positive values for ∆H^0^ and ∆S^0^ represent hydrophobic interactions. If the value of ∆H^0^ approaches zero and a compound has a positive ∆S^0^ the involvement of electrostatic forces is suggested [25,26]. The van’t Hoff plot for BSA and **6P** is represented in Figure 4D and the values for ∆G^0^, ∆H^0^ and ∆S^0^ are presented in Table 2. (−) ∆G^0^ indicates that BSA-**6P** binding was spontaneous. The calculated values for ∆H^0^ and ∆S^0^ were negative (–) indicating that the interaction between **6P** and BSA was enthalpy driven with unfavorable entropy and van der Waals forces and hydrogen bonding play main roles in the **6P** and BSA interaction. 

### 2.6. Synchronous Fluorescence Spectroscopy of BSA and ***6P*** Complex

**Figure 5:**
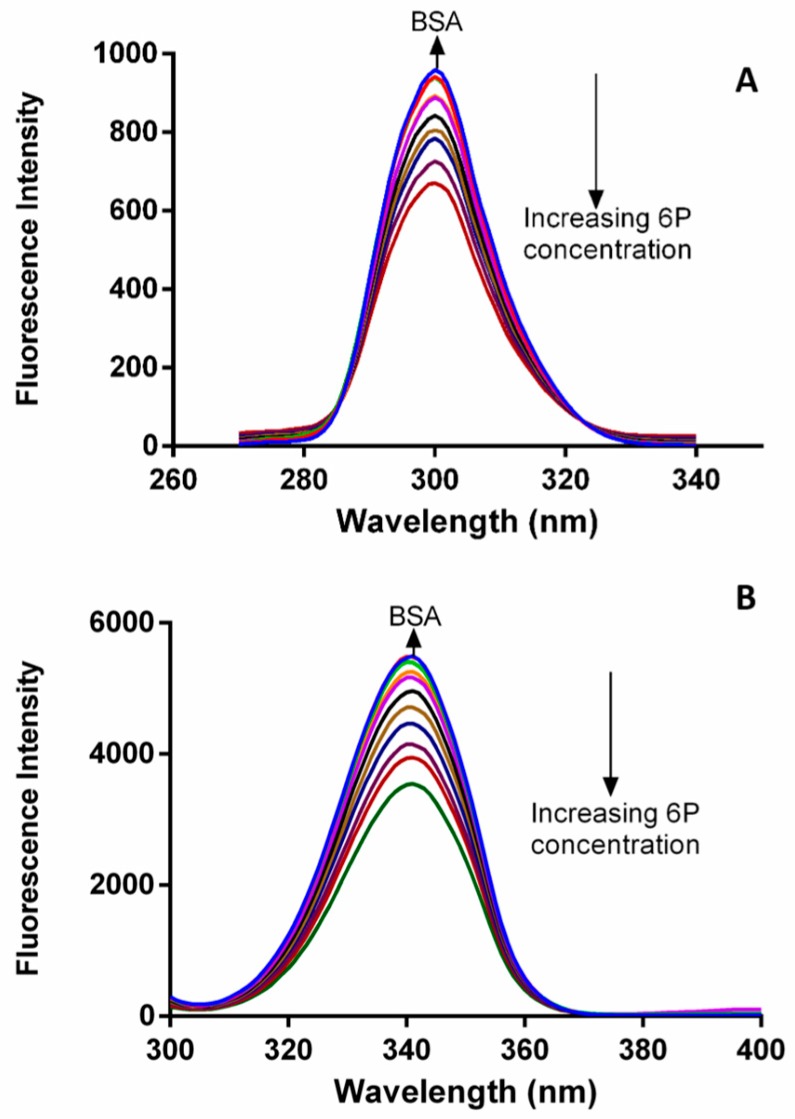
Synchronous fluorescence spectroscopy spectra of BSA at 298 K (**A**) ∆λ = 15 nm and (**B**) ∆λ = 60 nm.

Synchronous fluorescence spectroscopy (SFS) was used to study the microenvironment of the formed complex due to the **6P**-BSA interaction. SFS acts as an efficient tool to check the chromophore micro-environment [27]. The evidence regarding the tyrosine and tryptophan residue micro-enviroment is generated using scanning intervals of ∆λ = 15 nm and ∆λ = 60 nm, respectively. A shift in the emission wavelength of BSA suggests a change in the micro- environmental polarity of either tyrosine or tryptophan or both. The SFS of BSA were obtained without and with different concentrations of **6P** at both ∆λ = 15 nm and ∆λ = 60 nm (Figure 5). A shift of 1 nm in the emission wavelength was observed at ∆λ= 60 nm. This shift indicates a change in the micro-environment of tryptophan residue of BSA upon interaction with **6P**.

**Figure 6:**
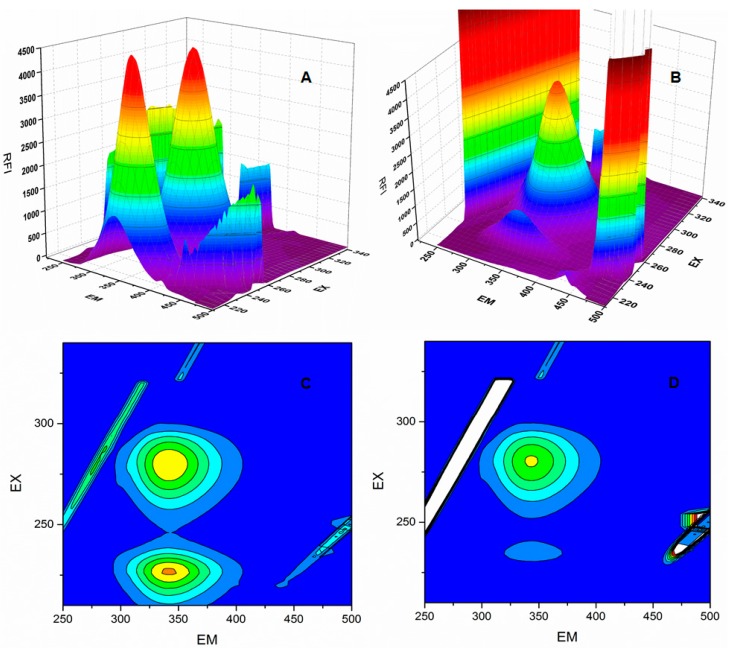
Three-dimensional fluorescence (3D) spectra and contour spectra of BSA (**A)** and (**C**) and BSA–**6P** (**B)** and (**D**) complex BSA.

The 3D (3-dimensional) spectra for BSA (Figure 6) were obtained with and without **6P** [28]. Peak 2 (λ_ex_/λ_em_: 280.0/344.0 nm) represents the presence of tryptophan and tyrosine residues and shows a decrease in the FI post **6P** addition. Peak 1 (λ_ex_/λ_em_: 234.0/344.0 nm) represents the polypeptides present in the BSA and is formed due to π‒π* transitions in the polypeptide structures present in BSA. A sharp decrease in the FI is observed post **6P** addition. Further, the contour plot shows sparse spectra in presence of **6P** compared to BSA alone, suggesting conformational changes in BSA post **6P** interaction.

### 2.7. Molecular Simulation Studies

To extract more information regarding the BSA-**6P** interaction docking studies were performed. The most favored binding site and the binding mode were determined using docking analysis. The two ligand binding sites (Site I and Site II) present on the BSA molecule characterize the hydrophobic grooves present in sub-domains IIA and IIIA, respectively.

**Figure 7:**
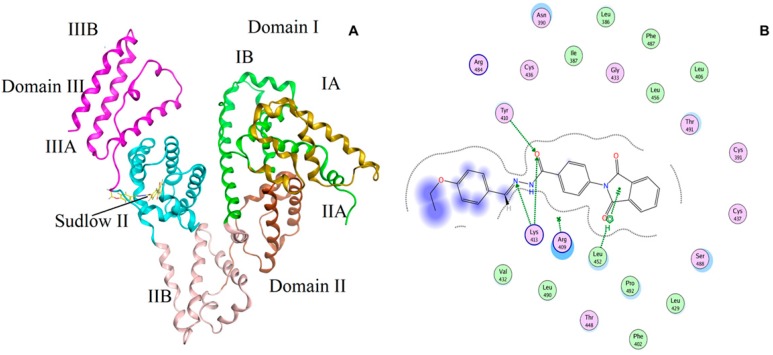
(**A**) The docking conformation of **6P**-BSA complex with lowest energy; (**B**) The amino acid residues surrounding **6P**.

Figure 7A presents the best conformation of **6P** and BSA complex and is clear that **6P** binds to site II of sub-domain IIIA of the BSA molecule. The docking results complimented the results generated using UV–visible spectroscopy and fluorescence spectroscopy. Figure 7B presents the hydrogen bonds involved between interactions of BSA to **6P**. Hydrogen bonds were formed at site II between **6P** and TYR-410 and LYS-413. The following amino acid residues ARG- 409, LEU-452, VAL-342, ARG-484, CYS-436, VAL-432, LEU-490 and THR-448 encircled **6P** and the calculated binding energy of BSA-**6P** complex was found to be 29.9 kJ·mol^−1^.

## 3. Materials and Methods

### 3.1. Chemical and Reagents

BSA was purchased from Sisco Research Laboratories (Mumbai, India). **6P** was synthesized in-house in the Medicinal Chemistry Department (m.p.: 248–250 °C; IR (KBr): *ν* max/cm^–1^: 3420 (CONH), 2927 (N=CH), 1739 (C=O); ^1^H-NMR (DMSO-*d*_6_) δ ppm: 1.3 (3H, t, CH_3_), 4.0 (2H, q, *J* = 5.5 Hz, -OCH_2_), 7.0–8.0 (12H, m, Ar-H), 8.4 (1H, s, N=CH), 11.8 (1H, s, CONH, D_2_O exchg.); ^13^C-NMR (DMSO-*d*_6_) δ ppm: 15.0, 63.7, 115.2, 124.0, 127.1, 127.5, 127.7, 128.6, 128.8, 129.1, 129.2, 131.9, 135.1, 135.3, 148.4, 162.8, 167.2; ESI mass (*m*/*z*): 412.10 [M − 1] ^+^ (calculated 413.42)). Phenylbutazone and ibuprofen was procured by from the National Scientific Company (Riyadh, Saudi Arabia). All the chemicals used for the study were of analytical grade. Phosphate buffer with pH 7.4 was used for the preparation of 1.5 µM BSA standard solution. **6P** stock solution was prepared by dissolving 5 mg in 500 in µL dimethyl sulphoxide and the volume was made up with phosphate buffer to get a concentration of 2.3 × 10^−3^ M. The working standards for **6P** ranged between 1.45 × 10^−6^ and 5.2 × 10^−5^ M were prepared using the stock. The site marker stocks phenylbutazone and ibuprofen were prepared in methanol and dilutions for them were made with phosphate buffer. An Elga Purelab system (High Wycombe, UK) was used to obtain type IV water for the preparation of solutions. 

### 3.2. UV Spectra Measurements

Spectrophotometric measurements were carried using a UV-1800 spectrophotometer (Shimadzu, Kyoto, Japan). These UV spectra were obtained for BSA alone and with variable concentrations of **6P**. All the measurements were carried out at room temperature.

### 3.3. Fluorescence Measurements

Fluorimetric spectra were obtained using a FP-8200 spectrofluorimeter (JASCO, Cremella, Italy). The spectra were obtained at excitation\emission wavelength of 280/340 nm respectively at temperatures of (298\303\308 K). The standard solutions were prepared by mixing fixed concentration of 1.5 × 10^−6^ M BSA solution in (1:1) *v*/*v* ratio with varied concentration 1.45 × 10^−6^ and 5.2 × 10^−5^ M of **6P** in 10 mL volumetric flasks and the final concentrations obtained for measuring the spectra were 0.75 × 10^−6^ M for BSA and 7.26 × 10^−7^ and 2.6 × 10^−5^ M for **6P**. The spectral measurement was repeated thrice and the final reading was the mean of the three experiments performed. The following equation was used to correct the inner filter effect of the fluorescence intensity.

(5) $$F_{\mathrm{cor}}=F_{\mathrm{obs}}\times e^{\left(A_{\mathrm{ex}}+A_{\mathrm{em}}\right)/2}$$

In the above equation F_cor_ represents the corrected fluorescence and F_obs_ represents the observed fluorescence. A_ex_ represents the absorption by **6P** at excitation wavelength and the A_em_ represents the **6P** absorption at emission wavelength. Since there is a possibility that the excitation and the emission wavelength can lie in the UV-absorption region, thus making it important to correct the inner filter effect.

### 3.4. Synchronous Fluorescence (SF) Measurement

A JASCO spectrofluorimeter was used for obtaining the synchronous fluorescence data. The data was obtained at room temperature using increasing concentrations of **6P**. The scanning intervals used for the analysis were ∆λ = 15 nm and ∆λ = 60 nm which correspond to the characteristics of tyrosine and tryptophan residues.

### 3.5. Molecular Docking

Molecular docking procedures provide a platform to analyze the interactions between ligands and proteins and support the experimental results. Docking analysis of the complex of **6P** and BSA was performed to observe the interaction of **6P** at the active binding site of BSA with the Molecular Operating Environment (MOE) suite docking software. For the docking analysis the crystalline structure of BSA was obtained from Protein Data Bank (http://www.rcsb.org) whereas the structure of **6P** was drawn inside the MOE software itself. The best configuration of BSA with **6P** was selected on the basis of RMSD (root mean square deviation) parameters.

## 4. Conclusions

**6P** being a promising anti-inflammatory drug was investigated for its interactions with the protein BSA in-vitro. **6P** was found to interact with BSA at site II in the subdomain IIIA of the BSA molecule. The complex formation was further explained with help of fluorescence quenching studies, spectrophotometric experiments, SFS, three dimensional fluorescence data and molecular modelling. A single binding site was observed on BSA to interact with **6P**. The complex formed between **6P** and BSA was enthalpy driven, with van der Waals forces and hydrogen bonding interactions playing the main roles. Serum albumin acts a carrier of drug ligands, thus, the results from our study will help in predicting the distribution and metabolism of **6P** in vivo, which would help us to further understand the pharmacodynamics of the **6P**. In addition this study provided vital information regarding the pharmacology and biochemistry of **6P** at the screening stage.

## Figures and Tables

**Table 1:** Stern–Volmer quenching constants (K_SV_) and bimolecular quenching rate constant (K_q_) for the binding of **6P** to BSA at three variable temperatures.

T (K)	R	K_sv_ (L·mol^–1^)	K_q_ (L·mol^−1^·s^−1^)
298	0.9934	2.73 × 10^4^	2.73 × 10^12^
303	0.9957	3.02 × 10^4^	3.02 × 10^12^
308	0.9913	3.20 × 10^4^	3.20 × 10^12^

**Table 2:** Binding and thermodynamic parameters of binding between **6P** and BSA.

T (K)	R	K_b_ (L·mol^−1^)	n	∆G (kJ·mol^−1^)	∆H (kJ·mol^−1^)	∆S (J·mol^−1^·K^−1^)
298	0.9980	2.5 × 10^5^	1.21	−31.01	−253	−744
303	0.9955	6.2 × 10^4^	1.06	−27.29		
308	0.9940	4.8 × 10^3^	0.81	−22.08		
